# Improving quality in national reference laboratories: The role of SLMTA and mentorship

**DOI:** 10.4102/ajlm.v3i2.200

**Published:** 2014-09-16

**Authors:** Rosemary A. Audu, Catherine C. Onubogu, Nkiru N. Nwokoye, Eke Ofuche, Shirematee Baboolal, Odafen Oke, Elizabeth T. Luman, Emmanuel O. Idigbe

**Affiliations:** 1 Human Virology Laboratory, Nigerian Institute of Medical Research, Nigeria; 2 National Tuberculosis Reference Laboratory, Nigerian Institute of Medical Research, Nigeria; 3 AIDS Prevention Initiative in Nigeria, Nigeria; 4 American Society for Microbiology, United States; 5 US Centers for Disease Control and Prevention, Nigeria; 6 US Centers for Disease Control and Prevention, United States

## Abstract

**Background:**

The Nigerian Institute of Medical Research houses two reference laboratories: the virology and tuberculosis laboratories. Both were enrolled in the Strengthening Laboratory Management Toward Accreditation (SLMTA) programme.

**Objective:**

To describe the impact of SLMTA and discuss factors affecting the results, with an emphasis on mentorship.

**Methods:**

The SLMTA programme was implemented from April 2010 through November 2012. Participants attended three workshops and executed quality improvement projects; laboratory auditors evaluated performance using a standard checklist. The virology laboratory did not receive mentorship; however, the tuberculosis laboratory had an international mentor who visited the laboratory four times during the programme, spending two to four weeks embedded within the laboratory during each visit.

**Results:**

There was an overall improvement in the performance of both laboratories, with the virology laboratory increasing 13% (from 80% at baseline to 93% at exit audit) and the tuberculosis laboratory increasing 29% (from 66% to 95%). These scores were maintained nine months later at the surveillance audit.

**Conclusion:**

The SLMTA programme resulted in improved and sustained quality management performance for both laboratories. Mentoring was a possible factor in the substantial improvement made by the tuberculosis laboratory and should be considered in order to augment the training received from the SLMTA workshops.

## Introduction

The level of implementation of laboratory standards in the African region, verified through the process of accreditation, has historically been very low.^1^ Until recently, most laboratories in Africa have not emphasised quality management systems (QMS) in the provision of healthcare services. In addition, lack of staff training and education, poor physical infrastructure, climate extremes and financial constraints^2^ have limited implementation of laboratory quality systems. The absence of National Laboratory Strategic Plans to provide roadmaps for the implementation of quality laboratory services, as well as the lack of National Laboratory Quality Standards to guide the provision of quality clinical laboratory services and accreditation in Nigeria, are also obstacles for the implementation of quality laboratory systems. An earlier study has also reported that the culture of QMS is uncommon in Nigerian laboratories;^3^ as such, there is a need to build this culture.

To strengthen the laboratory systems of African countries in a systematic approach, the US Centers for Disease Control and Prevention (CDC), in collaboration with the American Society for Clinical Pathology, the Clinton Health Access Initiative and the World Health Organization’s Regional Office for Africa (WHO AFRO), launched the Strengthening Laboratory Management Toward Accreditation (SLMTA) training programme in 2009.^4^ This programme, which focuses on strengthening laboratory management to achieve immediate laboratory improvement and accelerate accreditation preparedness, has been implemented in 47 countries worldwide and is expanding rapidly.^5^ The programme includes workshops, improvement projects, site visits and, in some cases, mentoring.^6^ Nigeria embraced the SLMTA programme in 2009 and, by 2010, seven laboratory experts were trained to roll out the SLMTA programme in 24 of the 344 Nigerian laboratories supported by the US President’s Emergency Plan for AIDS Relief (PEPFAR).

The Nigerian Institute of Medical Research (NIMR) has two reference laboratories, namely, the Human Virology Laboratory (virology laboratory) and the National Tuberculosis Reference Laboratory (TB laboratory), under its mandate ‘to conduct basic, applied and operational research for the prevention and control of communicable and non-communicable diseases of public health importance in the country in collaboration with the federal and state ministries of health and other stakeholders’.^7^ Both laboratories were amongst the 24 enrolled in the SLMTA programme. The two laboratories are similar in their institutional management and were supported by the same PEPFAR implementation partner. However, the TB laboratory was assigned a mentor to assist with SLMTA implementation, whilst the virology laboratory was not. We describe the impact of the SLMTA programme and discuss potential factors affecting the results, with an emphasis on mentorship.

## Research method and design

### Implementation sites

The virology laboratory has provided laboratory services to the Nigerian HIV treatment programme since 2002 and similar services for the PEPFAR HIV treatment project which commenced in 2004. The laboratory has the following sections: serology, immunology, chemistry, haematology and molecular diagnostics, which includes resistance testing for HIV. A total of 81 758 tests were performed by the virology laboratory in 2010. The laboratory staff comprised 14 degree-holding professionals, one diploma-holding professional, one certificate-holding professional, two data clerks, two phlebotomists, two cleaners, one driver and eight other administrative staff. This laboratory had some previous experience implementing QMS and, in 2008, had received International Organization for Standardization (ISO) 9001 accreditation,^3^ a general organisational certification of management processes. In preparing medical laboratories for international accreditation, the SLMTA programme employs ISO 15189, which specifies standards for QMS particular to medical laboratories. To help it meet these standards, which are more relevant to clinical laboratories, the virology laboratory enrolled in the SLMTA programme.

The TB laboratory was established to meet the institute’s research mandate. From 2005, the scope of services increased with inclusion of a ‘directly observed treatment short-course’ (DOTS) diagnostic centre, which brought about the expansion and renovation of the laboratory in order to meet the TB diagnostic service needs of both the private and public sectors. With the DOTS centre, many more TB suspects were referred to the laboratory for diagnosis, treatment and follow-up, and the diagnostic workload increased dramatically. The laboratory was commissioned as a National TB Reference Laboratory in 2007 and offers the following services: smear microscopy for acid-fast bacilli; solid and liquid culture; identification of *Mycobacterium tuberculosis* complex and Mycobacteria other than tuberculosis (MOTTs); and drug susceptibility testing using solid, liquid and molecular-based techniques. The laboratory is also involved in national TB drug resistance surveillance. A total of 80 799 tests were conducted in 2010. During the course of the SLMTA programme, the TB laboratory included 14 degree-holding staff, four diploma-holding staff, three microscopists, one administrative staff, one data clerk and one cleaner. Each laboratory also had a director, laboratory manager, quality assurance officer and dedicated personnel who had consistent job responsibilities throughout the duration of the programme.

### SLMTA implementation

**FIGURE 1:**
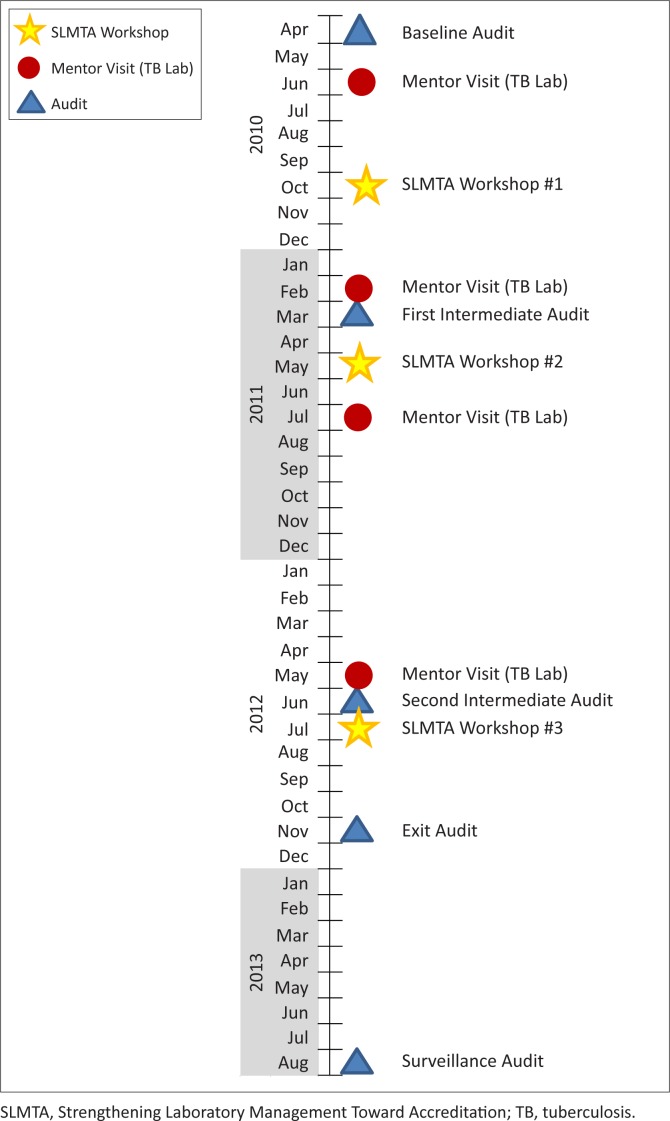
SLMTA implementation timeline in Nigerian reference laboratories.

The SLMTA programme was implemented in the NIMR virology and TB laboratories over two years and seven months (Figure 1). Three workshops were conducted within this period, with an average eight-month interval between them. The laboratory managers and quality assurance officers from both laboratories attended the workshops, after which they trained the other laboratory staff.

### Evaluation of laboratory performance

A baseline audit was conducted in each laboratory in April 2010, six months before the first SLMTA workshop (Figure 1). Intermediate audits were conducted after each workshop in order to monitor progress and to help identify any remaining gaps. An exit audit was conducted in November 2012, four months after the third workshop and a surveillance audit was conducted in August 2013, nine months after the exit audit, in order to determine the ability of the laboratories to sustain the quality advances made. The baseline and first intermediate audits were conducted using the laboratory accreditation preparedness checklist developed in 2009 by WHO AFRO.^8^ The checklist had a total score of 250 points distributed into 12 sections corresponding to the 12 quality system essentials. In 2012, WHO AFRO revised the checklist by adding more details in the requirements for documents and records and management review, as well as modifying the sectional scores, with a new total of 258 points.^9^ This revised checklist was used in parallel with the older version for the second intermediate and surveillance audits to allow us to evaluate the effect of the revision; results presented are from the revised checklist. For the exit audit, only the revised checklist was used. Based on the audit scores, laboratories were assigned a zero- to five-star rating, whereby < 55% = zero stars, 55% − 64% = one star, 65% − 74% = two stars, 75% − 84% = three stars, 85% − 94% = four stars and 95% − 100% = five stars. Independent laboratory experts who had taken the SLMTA training-of-trainers course, which included one day of training on auditing, were engaged as auditors; the same team conducted the audits in both laboratories, although different auditors were scheduled for each round of audit.

### Quality improvement projects

Quality improvement projects were selected by each laboratory after the first and second workshops based on laboratories’ specific needs within topics addressed at the workshops. The projects were implemented by all staff members and were monitored for effectiveness by their supervisors using the WHO AFRO checklist; reports were presented at the next workshop by the quality managers.

The virology laboratory embarked on three quality improvement projects after the first workshop. The first was that staff were trained on the importance of monitoring the autoclave with emphasis being placed on effective sterilisation and proper waste segregation. They were then assessed daily by means of an in-house-developed checklist in order to improve competency regarding sterilisation and waste disposal. In addition, provision was made for stock level on inventory cards, expired reagents were disposed of and general improvements were made to the organisation of the store; and the storage media for documents were monitored monthly to ensure ease of retrieval of records, documents and policies. After the second workshop, another set of improvement projects was conducted: complaint types, root causes, corrective actions and effectiveness were monitored in order to improve customer satisfaction; specimens in and out of the laboratory were clocked for three months to measure and improve turnaround time; and the number and duration of items out of stock were monitored in order to reduce stock-outs of materials, kits and reagents. The officer responsible for storage monitored the stock-outs from the weekly requisition and issue records.

For the TB laboratory, two quality improvement projects were implemented after the first workshop: monitoring inventory of reagents in order to improve turnaround time for acid-fast bacilli smear microscopy; and training staff on how to conduct internal audits. The laboratory conducted three improvement projects after the second workshop: monitoring media preparation and reviewing sputum-collection records in order to reduce the contamination rate of cultured samples; administering and analysing questionnaires from clients and effecting corrective actions in order to improve customer satisfaction; and improving documentation of inventory and establishing a requisition system for the stores.

### Mentorship and additional support

Contrary to SLMTA’s implementation roadmap,^5^ time constraints on the laboratory experts who rolled out the SLMTA programme in Nigeria prevented follow-up site visits at the virology laboratory between the workshops, which would have assisted in linking the training curriculum with on-site activities.

For the TB laboratory, an experienced international mentor from the American Society for Microbiology was assigned to work with the laboratory throughout the SLMTA process. Only TB laboratories were assigned mentors in this round of the SLMTA programme in Nigeria. The mentor had a postgraduate degree in quality management systems and a doctoral degree in microbiology with a TB specialty, had previously managed a laboratory that successfully attained international accreditation and had attended a SLMTA training-of-trainers workshop. A facility-based approach was adopted as the mentor visited the laboratory on four occasions for an average duration of three weeks at a time, allowing an in-depth understanding of the laboratory. The mentor provided daily assistance to the staff in the implementation of QMS, which included training in practical skills, assisting in improving quality of testing and giving assignments to be completed between visits. Nonconformities reported from each audit were addressed by the mentor at each visit and management review meetings were established to identify opportunities for improvement and to formulate action plans. The mentor had administrative support from institutional management and the Federal Ministry of Health, as well as technical and logistical support provided by CDC’s office in Nigeria.

Within the time frame of the SLMTA programme, the AIDS Prevention Initiative in Nigeria organised a five-day training on accreditation preparedness, with emphasis on quality management systems. Staff from both of the laboratories participated alongside staff from other laboratories that the organisation supports.

## Results

### Overall performance

**FIGURE 2:**
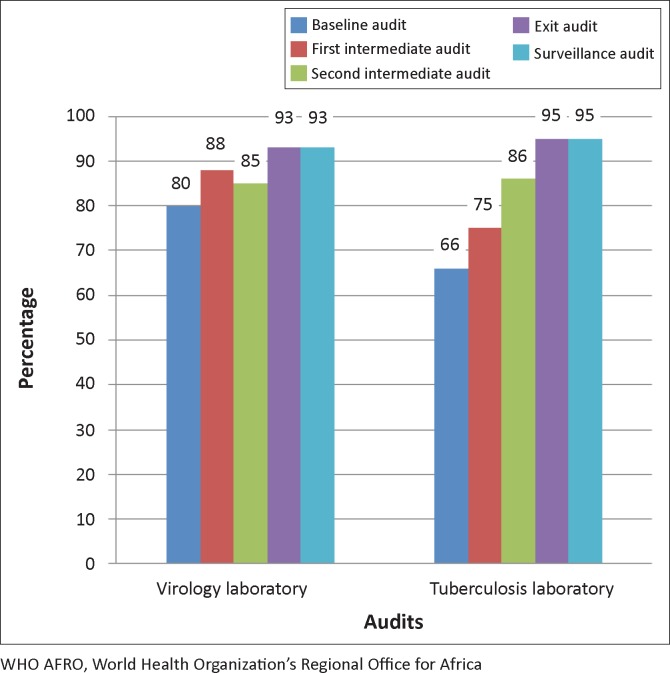
Comparison of performance of virology and tuberculosis laboratories over time using the WHO AFRO checklist.

There was an overall improvement in the performance of both laboratories during the SLMTA programme (Figure 2). The virology laboratory moved from an overall score of 80% at baseline, representing three stars, to 93% at the exit audit, representing four stars. The TB laboratory improved steadily from 66% at baseline audit, representing two stars, to 95% at exit audit, representing five stars. Both laboratories maintained these gains at the nine-month surveillance audit.

### Performance of quality system essentials

**FIGURE 3:**
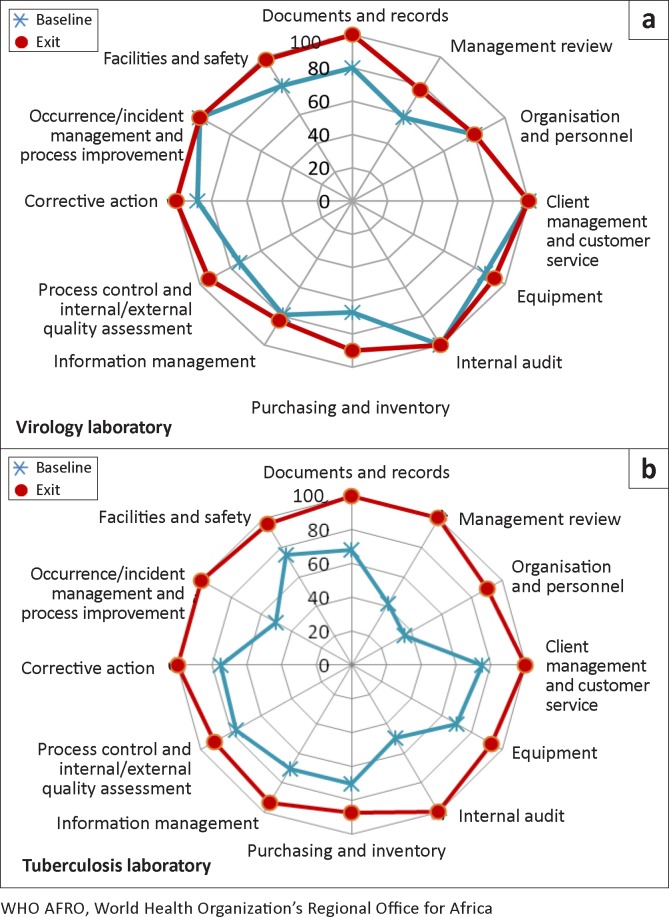
Comparison of performance of the virology and tuberculosis laboratories in the 12 quality system essentials using the WHO AFRO checklist.

Examining the 12 quality system essentials closely revealed specific areas of strength, weakness and improvement (Figure 3). The greatest improvements for the virology laboratory were in purchasing and inventory (from 67% to 90%) and in process control and internal and external quality assessment (from 74% to 94%) (Figure 3a). The virology laboratory achieved 100% scores in five quality system essentials (documents and records; client management and customer service; internal audit; corrective action; occurrence and/or incident management and process improvement); however no progress was made in organisation and personnel, which remained at 80% for the exit audit.

The TB laboratory generally started with lower scores than the virology laboratory, leaving more room for improvement. It made substantial improvements in management review (from 42% to 100%); internal audit (from 50% to 100%) and occurrence/incident management and process improvement (from 50% to 100%) (Figure 3b). The TB laboratory also had very good performance at the exit audit in all the quality system essentials, obtaining 100% scores in six sections (documents and records; management review; client management and customer service; internal audit; corrective action; and occurrence/incident management and process improvement).

### Quality improvement projects

The impact of the quality improvement projects on the quality system essentials is shown in Table 1. For the virology laboratory, after the first workshop the projects on documents and records and on purchasing and inventory impacted positively at the first intermediate audit and progress was sustained through the exit audit. The project on organisation and personnel did not have a positive impact on the score for the corresponding quality essential, as there was a drop in the next audit score, which did not improve beyond baseline at the exit audit. Of the projects conducted after the second workshop, only the client management and customer service project corresponded to sustained performance, with audit scores remaining at 100%, as they were at the baseline audit. Performance in information management was maintained at 93% after the second intermediate audit, but dropped to 83% at the exit audit, whilst purchasing and inventory improved from 93% before implementation to 97% at the second audit, but dropped to 90% at the exit audit. At the surveillance audit, improvements were generally sustained, except for purchasing and inventory, which reverted to the baseline score of 67%.

**TABLE 1:** Impact of quality improvement projects on quality system essentials in the virology and tuberculosis laboratories of the Nigerian Institute of Medical Research.

Workshop†	Quality system essential	Improvement project	Goal of project	Audit score (%)				
				Baseline	First intermediate	Second intermediate	Exit	Surveillance
**Virology laboratory**								
Firs**t**	Organisation and personnel	Trained staff and assessed daily with a checklist	To improve staff competency for sterilisation and waste disposal	80	75‡	85	80	100
	Purchasing and inventory	Monitored inventory cards, expired reagents and store organisation	To improve the organisation of the store	67	93‡	97	90	67
	Documents and records	Monitored ease of retrieval of documents and policies	To organise the laboratory documents, policies and records	80	92‡	84	100	96
Second	Client management and customer service	Monitored complaint types, root causes, corrective actions and effectiveness	To improve customer satisfaction	100	88	100‡	100	100
	Information management	Clocked specimens in and out of the lab for three months	To improve turnaround time	79	93	93‡	83	100
	Purchasing and inventory	Monitored number and duration of stocked-out items	To reduce stock-out rate of materials, kits and reagents	67	93	97‡	90	67
**Tuberculosis laboratory**								
First	Information management	Monitored stock-out of reagents	To improve turnaround time of AFB microscopy	71	86‡	93	94	100
	Internal audit	Conducted internal audit	To improve competency of staff in auditing	50	20‡	60	100	100
Second	Process control and internal and external quality assessment	Monitored media preparation and reviewed sputum collection records	To reduce contamination rate of cultures	77	79	81‡	91	93
	Client management and customer service	Administered and analysed questionnaires from clients and effected corrective actions	To improve customer satisfaction	75	100	100‡	100	100
	Purchasing inventory	Improving documentation of inventory and establishing a requisition system for the stores	To improve performance in purchasing and inventory	70	77	70‡	87	89

† The first workshop was conducted after the baseline audit. The second workshop was conducted after the first intermediate audit;

‡ Scores obtained after implementing quality improvement projects.

AFB, acid-fast bacilli.

For the TB laboratory, after the first workshop there was an improvement in the performance of the information management section, which continued through to the exit audit, where it reached 94% (Table 1). The internal audit score dropped initially, but improved subsequently, attaining 100% at the exit audit. After the second workshop, there was a gradual improvement for process control and internal and external quality assessment to 91% at the exit audit; and maintenance of client management and customer service at 100%. Purchasing and inventory scores decreased initially following project implementation (from 77% to 70%), then increased to 87% at the exit audit. The surveillance audit showed sustained or improved performance in all areas.

### Effect of changes to the audit checklist

Parallel audit scores using the revised (2012) checklist were slightly lower than those using the original (2009) checklist (Table 2). Differences ranged from 0% to 7% and were smaller at the surveillance audit than at the second intermediate audit, where they resulted in a change of star category for both the virology and TB laboratories.

**TABLE 2:** Comparison of audit scores based on the original 2009 WHO AFRO checklist and the revised 2012 checklist.

Laboratory	Audit	Original checklist		Revised checklist	
		% Score	Stars	% Score	Stars
Virology laboratory	Second intermediate audit	85.2	4	78.3	3
	Surveillance audit	94.3	4	93.0	4
Tuberculosis laboratory	Second intermediate audit	86.0	4	82.2	3
	Surveillance audit	95.1	5	95.2	5

WHO AFRO, World Health Organization’s Regional Office for Africa.

## Discussion

Both the virology and TB laboratories successfully improved their quality scores, increasing by 13% and 29%, respectively. The virology laboratory started with more experience and higher scores at the baseline audit. However, improvement in the TB laboratory was steady and the exit score exceeded that of the virology laboratory. The two laboratories were from same institution and had the same management commitment and partner support, with similar test menu diversity, test volume and staff strength. The major difference between SLMTA implementation in the two laboratories was the presence of a facility-based laboratory mentor in the TB laboratory.

Other countries, such as Kenya and Botswana, have found similar results when implementing an accreditation-readiness programme, with mentored laboratories showing greater improvement than their non-mentored counterparts.^10,11,12^ Whilst conclusive evidence is lacking, as none of these programmes were designed as case-control studies (i.e., with mentors randomly assigned to laboratories), the combined anecdotal evidence strongly supports the benefit of such mentorship. Mentors who spend extended, well-structured periods in the laboratory working alongside the staff and helping participants to put quality improvement into practice through direct, daily coaching, can provide the needed support to fast-track laboratories toward quality improvement.

The laboratories faced several challenges with regard to SLMTA implementation. Firstly, whilst the laboratory checklist was used to help identify and correct problems, an understanding of some of the requirements was often a challenge, especially in the virology laboratory where an experienced mentor was not available to assist. Similarly, the virology laboratory reported challenges in interpreting ISO 15189 standard requirements and auditor recommendations. Secondly, though many of the quality improvement projects were implemented successfully and increased performance of the quality system essentials, some of these advances were not sustained, especially in the virology laboratory. The purchasing and inventory section was affected worst as some records were not maintained. Sustainability is a common concern for any improvement programme; once the intense focus of implementation ceases, special efforts and continued supervision are required so as to ensure that old habits do not return. It is possible that the root causes of the deficiencies were not properly identified and addressed. Nevertheless, improvements in total scores were sustained, suggesting that quality improvement overall was maintained.

Successful achievement of the four to five star levels reached by the two NIMR laboratories indicates a high level of laboratory functioning and gives credibility to the quality of the laboratory test results produced for improved healthcare services. The 22 other laboratories in Nigeria’s first SLMTA round had similarly impressive results, moving from an average baseline of 60% to 87% at exit audit; 16 of the laboratories achieved four to five stars.^13^ These successes inspired Nigeria to implement a second round of SLMTA in 2013 and to begin discussions regarding further national expansion of the programme. Because of the potential benefit of on-site mentorship, national experts are being trained in Nigeria to play this critical role.

### Limitations

Our observations are subject to several limitations. The first of these is that mentorship was not assigned randomly. Whilst factors that we examined, such as management support, laboratory size and testing volume, were similar for the two laboratories, there may have been other unobserved factors that could account for some of the differences. For example, the laboratories chose different quality improvement projects to implement between workshops. A report by Maina et al. suggests that internal audits (which were implemented by the TB laboratory after the first workshop) may be a catalyst for improvements in other areas, as conducting self-review can identify areas that need improvement.^14^ Whilst the virology laboratory was already conducting internal audits before SLMTA, the TB laboratory started with a 50% score in this area and increased to 100%, potentially helping to explain their improvement in other areas. The second limitation is that the checklists used for the baseline and exit audits were not exactly the same, potentially introducing bias in the results. Comparison of the scores obtained by the two checklists used in parallel at the second intermediate and surveillance audits revealed that the revised checklist produced slightly lower results than the original checklist, suggesting that our overall improvement results are possibly conservative. The final limitation is that the auditors engaged in this study had only undertaken one day of training on auditing, which is not adequate to fully qualify them as auditors. Whilst the use of a checklist helps to standardise the auditing process, some variability may have been introduced because of inexperience.

### Conclusion

The SLMTA programm was successful in improving the quality of the laboratory systems in these two laboratories, as evidenced by improved and sustained audit scores. The laboratory with expert on-site mentorship improved farther and steadier, achieving a score of five stars. Our results suggest that laboratories should consider using on-site mentorship in order to augment the impact of SLMTA in implementing quality improvement.
